# Relationship between polypharmacy, sarcopenia and drug classes in hospitalized older patients

**DOI:** 10.1007/s40520-026-03452-x

**Published:** 2026-07-08

**Authors:** Gorkem Turhan, Ahmet Yalcin, Busra Gokce, Volkan Atmis, Murat Varli

**Affiliations:** 1 Internal Medicine Department, Ankara University School of Medicine, Ankara, Turkey; 2 Geriatric Medicine Department, Ankara University School of Medicine, Ankara, Turkey

**Keywords:** Sarcopenia, Polypharmacy, Geriatrics, Angiotensin receptor blockers, Skeletal muscle

## Abstract

**Background:**

Sarcopenia and polypharmacy are common geriatric syndromes associated with adverse outcomes in older adults. Although previous studies have suggested a potential relationship between these conditions, the independent nature of this association remains unclear, particularly among hospitalized populations. Certain medication classes may also exert muscle-protective effects that could influence this relationship.

**Purpose:**

To examine whether polypharmacy is independently associated with sarcopenia in hospitalized older adults and to evaluate the relationship between specific medication classes and sarcopenia prevalence.

**Methods:**

This cross-sectional study included hospitalized patients aged ≥ 65 years. Sarcopenia was diagnosed according to EWGSOP2 criteria, skeletal muscle mass was assessed using bioelectrical impedance analysis (BIA), and polypharmacy was defined as the regular use of five or more medications. Medication classes with possible muscle-protective effects were identified through a targeted literature review. Multivariate logistic regression analysis was performed to assess independent associations.

**Results:**

A total of 206 patients were included (60% female; median age: 78 years). The prevalence of sarcopenia was 39.8%. Polypharmacy was common in both sarcopenic (87.8%) and non-sarcopenic (80.6%) patients (*p* = 0.33), and the number of medications did not differ significantly between groups (7 ± 3 vs. 7.87 ± 3; *p* = 0.15). Angiotensin receptor blocker (ARB) use was more frequent in the non-sarcopenic group (40% vs. 15%; *p* = 0.005). In multivariate analysis, ARB use remained independently associated with lower odds of sarcopenia.

**Conclusion:**

Polypharmacy was not independently associated with sarcopenia in hospitalized older adults. However, ARB use was significantly associated with lower likelihood of sarcopenia, suggesting a potential muscle-protective effect. Further longitudinal research is warranted to clarify causality.

## Introduction

 Sarcopenia, characterized by age-related decline in skeletal muscle mass and strength, is a prevalent geriatric syndrome associated with increased risk of falls, frailty, disability, hospitalization, and mortality. Its pathophysiology is multifactorial, involving chronic inflammation, hormonal changes, nutritional deficits, physical inactivity, and comorbid chronic diseases [1]. According to the revised criteria of the European Working Group on Sarcopenia in Older People (EWGSOP2), sarcopenia diagnosis requires the presence of both low muscle strength and low muscle mass [2].

Polypharmacy, typically defined as the concurrent use of five or more medications, is another common and clinically relevant condition in older adults. It is especially prevalent in hospitalized populations, where multimorbidity and functional decline are frequent [2, 3]. Polypharmacy has been associated with numerous adverse outcomes, including falls, cognitive decline, frailty, and potentially sarcopenia [4, 5].

The relationship between polypharmacy and sarcopenia remains controversial. Some studies have reported a positive association between the two conditions, while others have failed to find a significant link [6, 7]. Recent literature suggests a significant relationship between polypharmacy and sarcopenia in community-dwelling older adults, but only three studies have evaluated this relationship in hospitalized older adults, of which two found a significant association [8]. Lastly, the association of sarcopenia with polypharmacy and higher number of medications was evaluated in a 2023 meta-analysis and systematic review. In sarcopenic older adults, the mean number of medications was 1.39 higher, and the prevalence of polypharmacy was 65% greater compared to non-sarcopenic counterparts. Importantly, this association was statistically significant in community-dwelling and outpatient populations, but not among hospitalized patients or nursing home residents [9].

Preclinical and observational studies have demonstrated that some drug classes, such as angiotensin-converting enzyme inhibitors (ACEi), angiotensin receptor blockers (ARB), and dipeptidyl peptidase IV inhibitors (DPP-4), exhibit positive effects on skeletal muscle [10–12]. Older individuals commonly use these drugs, and individuals with polypharmacy may also use beneficial drugs for skeletal muscle. The presence of these drugs can decrease the risk of sarcopenia, even in individuals with polypharmacy. This can confound the relationship between polypharmacy and sarcopenia.

This study aims to determine whether polypharmacy is independently associated with sarcopenia in older hospitalized patients, and whether drugs that positively affect skeletal muscle modify this relationship.

## Materials and methods

### Study design and case selection

**Fig. 1:**
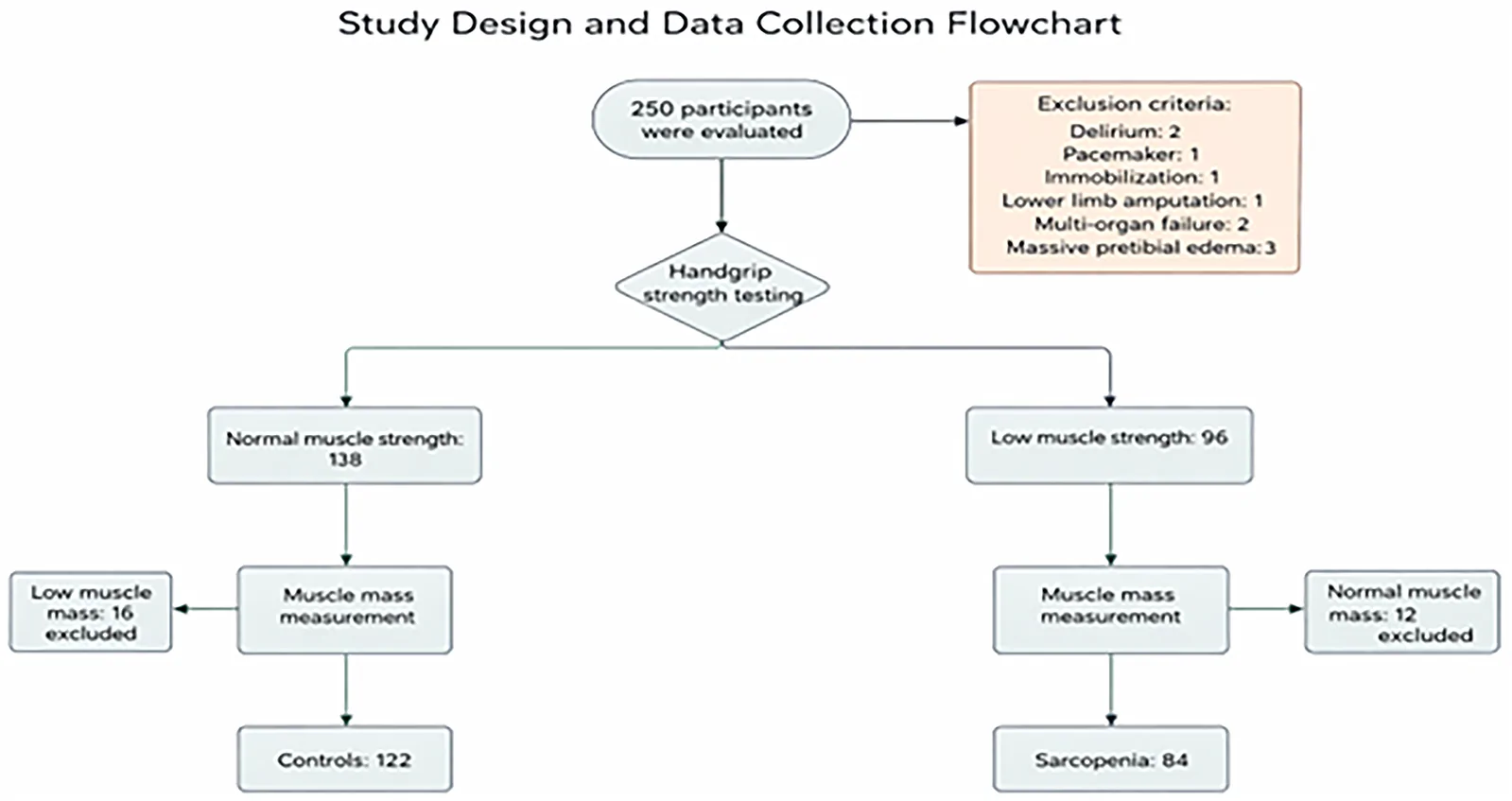
Study design and data collection flowchart

A cross-sectional study was conducted in the inpatient clinic of the Geriatric Medicine Department at Ankara University Faculty of Medicine İbn-i Sina Hospital (Ankara, Turkey). All older adults hospitalized for more than 24 h and willing to participate were assessed for participation into the study. Inclusion criteria were age ≥ 65 years and hospital stay > 24 h. Exclusion criteria were delirium or confusion, sepsis or multiple organ failure, immobilization, lower or upper limb amputation, massive pretibial edema, pacemaker presence, and unwillingness to participate. The study was approved by the Ankara University School of Medicine ethics committee. The data flow chart, including case selection and measurement steps, is shown in Fig. 1.

### Data collection, geriatric and sarcopenia assessment

All patients underwent an initial assessment within 48 h of admission. Sarcopenia is characterized by a progressive and generalized loss of skeletal muscle mass and strength, as defined by the European Working Group on Sarcopenia in Older People 2 (EWGSOP2).

A standardized form was used to gather demographic data, medical history, and medication history from the participants. Polypharmacy was defined as the daily use of five or more medications. The study focused on oral medications commonly prescribed to older adults, specifically those that have been shown to have beneficial effects on skeletal muscle. These included ACEi inhibitors, ARBs, biguanides, thiazolidines, incretins, allopurinol, β-agonists, statins, and vitamin D [13–16]. The Katz Activities of Daily Living (ADL) Index, a widely used measure of functional status, was employed to assess six basic ADLs: bathing, dressing, toileting, transferring, continence, and eating. A score of 6 on the Katz ADL Index indicates complete independence in ADLs, with increasing dependence associated with decreasing scores [17]. The Lawton-Brody Instrumental Activities of Daily Living (IADL) scale was administered to assess IADL performance. The scale comprises eight items, each scored 0 (dependent) or 1 (independent), yielding a total score ranging from 0 to 8. Higher scores indicate greater IADL independence [18]. Nutritional status was evaluated using the Mini Nutritional Assessment-Short Form (MNA-SF), a validated 14-point questionnaire. A score of 12–14 points indicates normal nutrition, while a score of 8–11 points suggests a risk of malnutrition. Scores of 0–7 points indicate malnutrition [19]. Cognitive status was evaluated using the Mini-Mental State Examination (MMSE), a 30-point instrument that assesses cognitive function across multiple domains, including orientation, registration, attention, recall, language, and visuospatial skills [20]. Gait speed (GS) was assessed twice using a stopwatch along a 4-meter marked walkway. The mean of the two GS measurements was calculated as the final GS value. A GS of ≤ 0.8 m/s was considered indicative of reduced GS [2].

Handgrip strength (HGS) was assessed using a Takei Scientific Instruments handheld dynamometer (Niigata, Japan) while participants were seated. The dynamometer was adjusted to fit each participant’s hand, and they were instructed to hold it with their proximal interphalangeal joints at 90 degrees of flexion and their elbows flexed at 90 degrees. Wrist support was provided if necessary. Participants were instructed to grip the dynamometer as forcefully as possible. Three grip strength measurements were obtained for each arm at one-minute intervals, and the highest value was recorded as skeletal muscle strength. Skeletal muscle strength was considered low if it was less than 16 kg in women and less than 27 kg in men. These cutoff values were based on the EWGSOP2 recommendations [21].

Skeletal muscle mass was determined using a Quadscan 4000 Bioimpedance Analysis (BIA) analyzer (Bodystat, Douglas, Isle of Man, United Kingdom). Prior to BIA evaluation, participant’s height, body weight, waist circumference, and hip circumference were measured. Measurements were taken at 08:00 h after participants had emptied their bladders and removed all metal jewelry. Electrodes were placed on the right ankle and wrist. Skeletal muscle mass was calculated using a previously validated formula incorporating the 50 Hz frequency value. Skeletal muscle mass was estimated using the formula proposed by Janssen et al. as follows [22].

$$\begin{aligned}\left( {\left( {{\text{Height }}\left( {{\mathrm{cm}}} \right){\text{ 2 }}/{\text{ BIA resistance value x }}0.{\mathrm{4}}0{\mathrm{1}}} \right)} \right){\text{ }} + \\{\text{ }}\left( {{\text{Gender x }}0.{\mathrm{825}}} \right){\text{ }} + {\text{ }}\left( {{\text{Age x }} - 00{\mathrm{71}}} \right)\, + \,{\mathrm{5}},{\mathrm{1}}0{\mathrm{2}}. \end{aligned}$$

In the formula, the gender variable was coded as 1 for males and 0 for females. Skeletal muscle mass was measured in kilograms. Absolute skeletal muscle index (ASMI), expressed in kilograms per square meter (kg/m2), was calculated by dividing the skeletal muscle mass by the square of the participant’s height in meters. A low ASMI (< 7 kg/m2 for males and < 5.5 kg/m2 for females) is indicative of low skeletal muscle mass. According to EWGSOP2 criteria, sarcopenia was diagnosed in participants with both low muscle strength and low muscle mass. Gait speed was assessed as a measure of physical performance and disease severity, but was not used as a diagnostic criterion.

### Laboratory assessment

Hemoglobin, C-reactive protein (CRP), and albumin were assessed as laboratory parameters in this study. These are common blood tests routinely performed during hospitalization.

### Statistical analysis

All statistical analyses were performed using SPSS software (version 25.0; IBM Corp., Armonk, NY, USA). Continuous variables were summarized using mean ± standard deviation for those determined to be normally distributed using appropriate normality tests. For continuous variables that did not exhibit a normal distribution, the median (interquartile range) was reported. Group differences in two normally distributed continuous variables were assessed using an independent-samples t-test. In the absence of normally distributed continuous data, the Mann-Whitney U test was utilized for comparative analysis. Categorical variables were summarized as percentages. The chi-square test was used to evaluate the presence of a statistically significant association between categorical variables. The Pearson and Spearman correlation coefficients were utilized to assess the correlation between continuous variables. Logistic regression analysis was used to identify factors associated with sarcopenia. Variables were incorporated into the multivariate analysis based on the findings of the univariate analysis. Odds ratios and 95% confidence intervals were calculated following adjustment for covariates. A p-value of less than or equal to 0.05 was considered statistically significant. Variables included in the multivariable model were selected based on both clinical relevance and univariate analysis findings to reduce the risk of model overfitting.

## Results

### Descriptive statistics

Participants with sarcopenia had significantly lower BMI, MMSE scores, and ASMI, and a higher prevalence of dementia, compared to non-sarcopenic participants (Table 1). The baseline characteristics of the participants are presented in Table 1.

**Table 1:** Clinical characteristics of study participants according to sarcopenia status

Variables	Sarcopenia (*n*:84)	Non-sarcopenic (*n*:122)	*p* value
Age (years) (mean ± SD)	80 ± 7.7	77.6 ± 7	**0.012**
Gender (%) Male Female	%39 %61	%33.9 %66.1	0.59
HT (%)	%38	%41	0.20
MI (%)	%22	%16.1	0.45
Heart failure (%)	%31.7	%24.2	0.4
PAD (%)	%12.2	%3.2	0.11
CVI (%)	%19.5	%14.5	0.5
Dementia (%)	%34.1	%11.3	**0.005**
COLD (%)	%22	%17.7	0.6
Diabetes (%)	%58.5	%58.1	0.96
CRD (%)	%48.8	%38.7	0.31
Cancer (%)	%4.9	%14.5	0.2
BMI (kg/m^2^) (mean ± SD)	25.4 ± 5	29.4 ± 6	**0.001**
Katz BADL (n) (median(IQR))	4(5)	5(3)	0.07
Lawton IADL (n) (median(IQR))	2.5(8)	5(6)	0.15
MNA-SF (n) (median(IQR))	10(5)	10(4)	0.5
MMSE (n) (median(IQR))	21(8)	24(9)	**0.026**
ASMI (kg/m^2^)	6.7(1.6)	9.5(2.7)	**< 0.01**
HGS (kg) (median(IQR))	10.2(9.55)	12.4(7.2)	0.07
GS (m/s) (median(IQR))	0.25(0.5)	0.31(0.38)	0.3
Albumin (g/dl) (median(IQR))	3.5(0.8)	3.5(0.62)	0.37
Hb (g/dl) (mean ± SD)	11.3 ± 2.1	11.1 ± 1.6	0.57
CRP (mg/dl) (median(IQR))	10(41.8)	12(56.8)	0.5

Standard derivation, Myocard infarction, Peripheral artery disease, Cerebrovascular incident, Chronic obstructive lung disease, Chronic renal disease, Body mass index, Basic activities of daily living, Inter quartile range, Instrumental activities of daily living, Mini nutritionl assessment- short form, Mini mental state examination, Absolute skeletal muscle index, Handgrip strength, Gait speed, Hemoglobin, C-reactive protein

### Polypharmacy and number of medications

Within the present study, 60.2% of individuals met the criteria for polypharmacy. The mean number of medications was 7.34 ± 3.01. No statistically significant difference in polypharmacy prevalence was observed between the sarcopenia group (%87.8) and the control group (%80.6), with a p-value of 0.33. In addition, no statistically significant difference in number of medications was observed between the sarcopenia group (7 ± 3) and the control group (7.87 ± 3), with a p-value of 0.15.

### Association between specific muscle parameters and number of medications

No significant correlation was observed between handgrip strength or ASMI and the number of medications. However, gait speed demonstrated a weak negative correlation with the number of medications, as demonstrated in Table 2.

**Table 2:** The correlation between number of drugs and sarcopenia components

Variables	Number of drugs	*p* value
Muscle strength	*r*:0.06	0.55
ASMI	*r*:-0.13	0.12
GS	*r*:-0.16	0.027

Absolute Skeletal muscle index, gait speed

### Presence of drug classes according to sarcopenia status

The only statistically significant difference between study groups by drug class was observed for ARBs. In participants with sarcopenia, angiotensin receptor blockers (ARBs) use was significantly **lower** compared to controls, with a prevalence of 15% versus 40%, respectively (*p* = 0.005) (Table 3). The associations between polypharmacy, drug classes, and sarcopenia are summarized in Table 3.

**Table 3:** The prevalence of drug classes according to sarcopenia status

Drugs	Sarcopenia	Controls	*p* value
Metformin (%)	%22	%24	0.8
ACE inhibitors (%)	%22	%13	0.22
ARBs (%)	%15	%40	**0.005**
DPP-4 inhibitors (%)	%34	%18	0.057
Sulfonylureas (%)	%5	%11	0.3
Statins (%)	%32	%29	0.78
Allopurinol (%)	%5	%5	1
β-agonists (%)	%19	%17	0.8
Vitamin D supplement (%)	%17	%8	0.2

Angiotensin converting enzyme, Angiotensin receptor blockers, Dipeptidyl peptidase

In univariate logistic regression analysis, age, BMI, dementia, ARBs, Katz BADL, MMSE and CC were significantly associated with the risk of sarcopenia. All significant variables, were included in the multivariate logistic regression analysis, age, ARBs and dementia remained significant (Table 4). The multivariate analyses of factors associated with sarcopenia are presented in Table 4.

**Table 4:** Factors associated with sarcopenia in logistic regression analysis

Variables	Univariate Analysis		Multivariate Analysis	
	OR (%95 CI)	*p* value	OR (%95 CI)	*p* value
Sex Male Female	Reference 1.25 (0.55–2.83)	0.59		
Age (years)	1.07(1.01–1.13)	0.015	1.02(0.96–1.09)	0.4
BMI (kg/m^2^)	0.87(0.8–0.95)	0.002	0.88(0.8–0.98)	0.02
CCI (n)	1.12(0.96–1.31)	0.15		
Diabetes No Yes	Reference 0.98(0.44–0.98)	0.96		
Dementia No Yes	Reference 4.07(1.47–11.27)	0.007	5.43(1.37–21.6)	0.016
COLD No Yes	Reference 1.3(0.49–3.49)	0.6		
CVD No Yes	Reference 1.42(0.5–4.07)	0.5		
MI No Yes	Reference 1.46(0.53–3.98)	0.46		
CRD No Yes	Reference 1.5(0.67–3.34)	0.31		
Cancer No Yes	Reference 0.3(0.06–1.48)	0.14		
Polypharmacy No Yes	Reference 1.73(0.56–5.34)	0.34		
Number of drugs (n)	1.1(0.96–1.26)	0.15		
Metformin No Yes	Reference 0.88(0.34–2.26)	0.79		
ACEi No Yes	Reference 1.89(0.66–5.41)	0.23		
ARBs No Yes	Reference 0.25(0.09–0.69)	0.007	0.18(0.05–0.66)	0.01
DPP4 inhitors No Yes	Reference 2.4(0.96–6.01)	0.061		
Vitamin D Supplements No Yes	Reference 2.35(0.69–7.98)	0.17		
Allopurinol No Yes	Reference 1(0.16–6.32)	0.1		
Β-agonists No Yes	Reference 0.0.86(0.3–2.4)	0.77		
Sulphonureas Yes No	Reference 0.4(0.08–2.04)	0.27		
Statins Yes No	Reference 1.14(0.48–2.67)	0.12		
Katz BADL (n)	0.82(0.67-1)	0.04	0.9(0.7–1.15)	0.4
Lawton IADL (n)	0.95(0.87–1.05)	0.32		
MNA-SF (n)	0.91(0.77–1.07)	0.26		
MMSE (n)	0.94(0.88-1)	0.08		
CC (n)	0.88(0.8–0.96)	0.005	1.01(0.87–1.18)	0.83
Hemoglobin (g/dl)	1.06(0.86–1.3)	0.56		
Albumin (g/dl)	0.65(0.3–1.41)	0.28		
CRP (mg/dl)	0.99(0.98-1)	0.64		
25-OH Vitamin D (ng/ml)	0.8(0.96–1.02)	0.8		

Odds ratio, Confidence interval, Body mass index, Charlson Comorbidity Index, Chronic Obstructive Lung Disease, Cerebrovascular disease, Myocard infarction, Chronic renal disease, Angiotensin converting enzyme inhibitors, Angiotensin receptor blockers, Dipeptidyl peptidase, Mini nutritional assessment-short form, Activities of daily living, Instrumental activities of daily living, Mini mental state examination, Calf circumference C- reactive protein

## Discussion

Sarcopenia and polypharmacy are prevalent geriatric syndromes affecting a significant proportion of the older adult population. This study investigated the association between polypharmacy and sarcopenia in hospitalized older adults, with a specific focus on how medications with reported benefits for skeletal muscle function may influence this association. Our findings revealed no link between sarcopenia and polypharmacy in hospitalized older adults. While the association between sarcopenia and polypharmacy has been explored in the literature, a definitive causal relationship remains to be elucidated. A systematic review of the literature published in 2022 was conducted to identify studies examining the association between polypharmacy and sarcopenia. A total of 22 studies were included. Among community dwelling older adults, sarcopenic individuals exhibited a higher number of medications and a greater prevalence of polypharmacy across all diagnostic criteria employed. This discrepancy was not observed among older adults residing in nursing homes or hospitalized. This situation may arise from various underlying factors. Variations in study populations, threshold values, and diagnostic criteria may have yielded difference in findings. In this systematic review, studies included community-dwelling older adults, nursing home residents, older adults in outpatient and hospital settings. Polypharmacy was defined as the concurrent use of four or more medications in two out of the 22 studies. In eight studies, the number of medications was specified without employing a definition of polypharmacy. The remaining studies classified polypharmacy as the use of five or more medications. Sarcopenia diagnosis criteria are most commonly based on EWGSOP and EWGSOP2 guidelines. Other employed diagnostic criteria were Cardiovascular Health Study, the Asian Working Group for Sarcopenia, and the Foundation of the National Institutes of Health criteria. Anthropometric measurements, bioelectrical impedance analysis (BIA), and dual-energy X-ray absorptiometry (DEXA) have been predominantly utilized for muscle mass assessments. Upon evaluation using both EWGSOP and EWGSOP2 criteria, no association was identified between polypharmacy or number of medications and sarcopenia, irrespective of the study population [8]. The utilization of EWGSOP2 criteria in our study may have been a reason for no association between sarcopenia and polypharmacy. Hospitalized older adults represent a clinically complex and heterogeneous population. Acute illness, inflammatory burden, reduced mobility, hydration changes, and nutritional deterioration may substantially influence muscle-related measurements including handgrip strength, gait speed, and bioimpedance-derived muscle mass estimates.

Agosta et al. evaluated the relationship between sarcopenia and polypharmacy using data from the multicenter GLISTEN study, which was conducted in Italy and investigated factors associated with sarcopenia in hospitalized older individuals. The diagnosis of sarcopenia was made according to EWGSOP criteria, and the definition of polypharmacy was defined as 5 or more drugs. The frequency of polypharmacy was found to be 59.9% in individuals with sarcopenia and 53% in individuals without sarcopenia. However, this difference was not found to be statistically significant. Similarly, no difference was found for hyperpolypharmacy [6]. In community-dwelling elderly individuals, polypharmacy has been associated with sarcopenia [7]. The authors of the GLISTEN study reported that the effect of polypharmacy on sarcopenia may be reduced if the participants were older, had more and more severe diseases, and had poor functional and nutritional status [6]. These results are compatible with the results of our study. Similarly, in our study, sarcopenic individuals were older and had a higher rate of dementia, and their MMSE scores and BMI values, which are an indicator of nutritional status, were lower. However, none of these studies evaluated the relationship between drug type and sarcopenia. Although metformin has been suggested to exert potentially beneficial metabolic and anti-inflammatory effects on skeletal muscle, caution is warranted in frail and malnourished older adults. The Turkish Inappropriate Medication Use in the Elderly (TIME) criteria emphasize that metformin may contribute to gastrointestinal adverse effects and reduced nutritional intake in vulnerable older individuals. Therefore, the relationship between metformin and sarcopenia may be complex and bidirectional [23, 24]. Although several medication-related differences did not reach statistical significance, the observed effect sizes may still be clinically meaningful given the relatively limited sample size and heterogeneity of hospitalized geriatric populations. In addition, although SGLT2 inhibitors have recently been suggested to potentially reduce lean body mass and skeletal muscle mass, the number of SGLT2 inhibitor users in our cohort was insufficient for meaningful subgroup analysis.

The efficacy of angiotensin receptor blockade on skeletal muscle has been validated through a plethora of experimental and clinical investigations. A group of researchers explores Losertan’s potential to combat sarcopenia in an experimental model. Researchers compared two groups of aged mice: one treated with with Losartan for four months and another receiving a placebo. Losartan showed significant improvements in activity levels compared to placebo. Losartan group were more active on treadmills, stood for longer periods, and fell less often by reducing inflammation and oxidative stress [25]. In another study, researchers investigated if ARBs could improve muscle function in COPD patients by affecting neuromuscular junction. The study involved 236 participants with COPD. The participants were divided into three groups: Normotensive, hypertensive taking ARBs, hypertensive taking other medications for hypertension. Researchers measured muscle strength, body composition, physical performance, and c-terminal fragment of agrin-22 (CAF-22) as a marker for neuromuscular junction degradation at the beginning and one year later. The hypertensive group taking ARBs showed improvements in HGS, GS and levels of CAF-22. The ARB group also showed no decline in physical performance. The study suggests that ARBs may help preserve muscle health and physical function in hypertensive patients with COPD [26].

Harada et al. explored the correlation between prescribed medications and skeletal muscle mass among 636 hospitalized participants, with a mean age of 73 years, diagnosed with cardiovascular disease. They assessed muscle mass using bioelectrical impedance analysis (BIA) and standardized the skeletal muscle index by dividing it by the square of the individual’s height in meters. Evaluated pharmaceutical agents include angiotensin II receptor blockers (ARBs), β-blockers, calcium channel blockers, statins, angiotensin-converting enzyme inhibitors (ACEIs), diuretics, antiplatelet and anticoagulant medications, dipeptidyl peptidase-4 (DPP-4) inhibitors, agents for hyperuricemia, antiarrhythmic drugs, as well as various combinations. Based on the findings of their multivariate analysis, it was determined that hyperuricemia medications, DPP-4 inhibitors, and combinations of ARB/statins exhibited a significant and independent correlation with skeletal muscle index. Diuretics and antiarrhythmic drugs were each independently observed to exhibit a negative association with skeletal muscle index [27].

A systematic review and meta-analysis, published in 2020, investigated the impact of ACEis and ARBs on physical performance in older adults. Randomized controlled trials involving older adults and comparing ACEi or ARB to placebo, usual care, or another blood pressure medication. Physical performance was measured by Short Physical Performance Battery. No significant improvement was observed with either ACEis or ARBs compared to controls. However, there was heterogeneity in the results. The discrepancy between ACE inhibitor and ARB findings in our study may be related to the relatively small sample size, limited statistical power, and potential mechanistic differences between renin-angiotensin system blockers.

### Limitations

The cross-sectional design of our study imposes a limitation on establishing causality. There is a need for large-scale, prospective, randomized controlled studies investigating the effects of ARBs on sarcopenia While our study intentionally excluded drugs such as chemotherapy agents or corticosteroids, it is essential to acknowledge another limitation regarding the absence of evaluation for other pharmaceuticals known to potentially exert adverse effects on skeletal muscle. Residual confounding related to acute hospitalization status and unmeasured disease severity cannot be excluded. Admission diagnoses and medication interactions may also have influenced the observed associations. Because this study was conducted in hospitalized older adults, acute illness severity and hospitalization-related factors may have influenced muscle-related measurements independent of chronic medication exposure.

## Conclusions

Sarcopenia and polypharmacy are prevalent and clinically relevant geriatric syndromes associated with adverse health outcomes in older adults. In this cross-sectional study of hospitalized older patients, no independent association was observed between polypharmacy and sarcopenia. However, the use of angiotensin receptor blockers (ARBs) was significantly associated with a lower likelihood of sarcopenia. Given the observational nature of this study, these findings should be interpreted as associations rather than causal effects. Further prospective, multicenter studies with larger cohorts are warranted to explore the potential role of specific drug classes in the prevention or mitigation of sarcopenia. The observed associations should be interpreted cautiously and considered hypothesis-generating rather than causal due to the cross-sectional nature of the study.

## Data Availability

No datasets were generated or analysed during the current study.
